# Clitoromegaly, Vulvovaginal Hemangioma Mimicking Pelvic Organ Prolapse, and Heavy Menstrual Bleeding: Gynecologic Manifestations of Klippel-Trénaunay Syndrome

**DOI:** 10.3390/medicina57040366

**Published:** 2021-04-09

**Authors:** Gina Nam, Sa Ra Lee, SeungA Choi

**Affiliations:** 1 Department of Obstetrics and Gynecology, Chung-Ang University Hospital, Chung-Ang University College of Medicine, 102, Heukseok-ro, Dongjak-gu, Seoul 06973, Korea; ginanam@caumc.or.kr; 2 Department of Obstetrics and Gynecology, Asan Medical Center, University of Ulsan College of Medicine, 88, Olympic-ro 43-gil, Songpa-gu, Seoul 05505, Korea; chsa1002@gmail.com

**Keywords:** Klippel-Trénaunay syndrome, clitoris, hemangioma, pelvic organ prolapse, heavy menstrual bleeding, whole genome sequencing

## Abstract

Klippel-Trénaunay Syndrome (KTS) is a genetic vascular malformation involving the capillary, lymphatic, and venous channels. Prenatal sonographic diagnosis of KTS with an enlarged fetal limb is well-known; however, postnatal gynecologic manifestations are rarely reported. KTS can cause clitoromegaly, vulvovaginal hemangioma, and heavy menstrual bleeding. Somatic mosaicism of the *PIK3CA* gene is considered as responsible for KTS but reports based on whole-genome sequencing are limited. A 31-year-old woman with KTS presented with bulging of the clitoris and vagina. Analysis of whole-genome sequencing variant data revealed that gene ontology terms related to development and differentiation such as ‘skeletal system morphogenesis’, ‘embryonic morphogenesis’, and ‘sensory organ development’ were nominally significant in non-coding regions. Variants in non-coding genes may be responsible for this phenotype.

## 1. Introduction

Klippel-Trénaunay syndrome (KTS) is a rare vascular malformation with an incidence of 1/30,000 live births [1]. It is defined as a triad of the following: (1) capillary lymphangiovenous malformations, which are typically presented as port-wine stains, a type of cutaneous capillary malformation or superficial vascular blebs, and a type of lymphatic malformation; (2) soft tissue and bone hypertrophy, beginning in infancy and most often only affecting one leg; and (3) varicose veins or venous malformation, mostly with persistent lateral embryologic veins [2]. KTS can be diagnosed when two of the three features are observed. Genitourinary involvement and postnatal gynecologic problems associated with KTS are rarely reported. Herein, we report the first case of clitoral and vaginal manifestations of KTS mimicking those of clitoromegaly and pelvic organ prolapse. Accompanying heavy menstrual bleeding (HMB) was well-controlled with non-steroidal anti-inflammatory drugs (NSAIDs). KTS is a sporadic congenital disorder resulting from somatic mutations of the phosphatidylinositol-4,5-bisphospate 3-kinase, catalytic subunit alpha (*PIK3CA*) gene, which is involved in cell growth and proliferation [3]. We also performed whole-genome sequencing (WGS) to detect novel KTS variants responsible for the vascular phenotype.

## 2. Case

**Figure 1:**
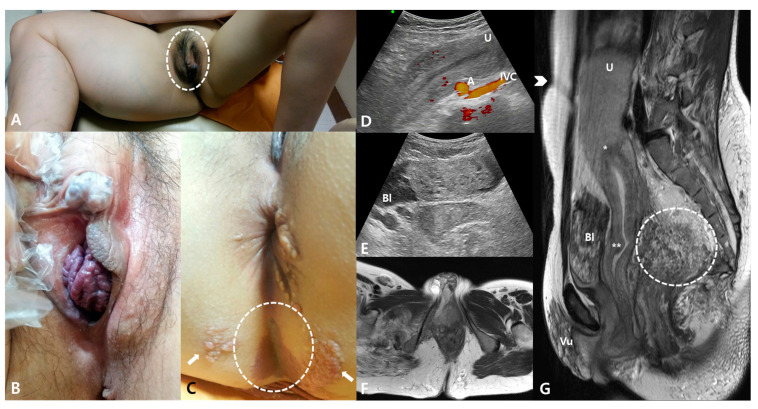
(**A**) Hemihypertrophy of the right lower limb, hemangioma of the right vulva, and ambiguous genitalia with an elongated clitoral hood, approximately 4 cm in length (shown in the dotted circle). (**B**) An enlarged, 2.0 × 1.8-cm^2^ whitish globular clitoris glans and a purplish bulging anterior vaginal wall. (**C**) Typical port-wine nevus (dotted circle) and bullous plaques (arrows) on the buttock. (**D**) The upper margin of the uterine fundus (U) was noted around the abdominal aorta (**A**) and inferior vena cava (IVC) on sonography. (**E**) A typical sponge-like shadow consisting of a mass-like lesion without Doppler flow in the bladder (Bl) was observed by sonography. (**F**) Hemihypertrophy of the right lower limb and hemangioma of the right vulva with an enlarged clitoris viewed with MRI. (**G**) A typical sponge-like shadow in the bladder (Bl) and rectum (dotted circle) viewed in T1-weighted images, and an extensive cavernous hemangioma in the pelvis pushing the uterine fundus (U) up to above the umbilicus (arrowhead) shown on MRI. * (internal cervical os); ** (external cervical os).

A 31-year-old woman with known KTS presented with a progressive bulging of the clitoris and vagina associated with aggravating HMB. A physical examination revealed hemihypertrophy of the right lower limb, hemangioma of the right vulva, and ambiguous genitalia with an elongated clitoral hood approximately 4 cm in length (Figure 1A). An enlarged, 2.0 × 1.8-cm^2^ whitish globular glans of the clitoris and a purplish bulging anterior vaginal wall mimicking pelvic organ prolapse were also observed (Figure 1B). Typical port-wine nevus, capillary malformation, and bullous plaques, pathologically proven lymphatic malformations, were detected on the buttock (Figure 1C). Sonography and magnetic resonance imaging (MRI) showed a typical sponge-like shadow in the vulva, bladder, and rectum on T1 weighted images and an extensive cavernous hemangioma in the pelvis pushed the uterine fundus up to the above the umbilicus (Figure 1D–G). The upper margin of the uterine fundus was observed around the third lumbar spine vertebra. The HMB was well-controlled with oral aceclofenac, an NSAID, during menstrual periods.

A genetic analysis by WGS was performed using peripheral blood DNA on an Illumina HiSeq X system (Illumina, San Diego, CA, USA). The average read length was 150 base pairs. DNA was prepared according to the Illumina DNA sample preparation guide. For alignment and genotyping, an Isaac aligner (Illumina, San Diego, CA, USA) and a variant caller (Illumina, San Diego, CA, USA) were applied. The hg19 reference genome from the University of California Santa Cruz (UCSC) genome database was used. 

### 2.1. Identification of Mutation Variants Associated with KTS

To detect variants responsible for the development of KTS, we used the wANNOVAR (http://wannovar.wglab.org/ (accessed on 9 February 2021)) (Wang Genomics Lab, Philadelphia, PA, USA). The wANNOVAR is a web version of the ANNOVAR (ANNotation of VARiation) database used for the interpretation and prioritization of variants in a genome. The variants include single-nucleotide variants, insertions, deletions, and copy number variants. After annotation, the variants can be filtered according to the criteria relevant to the disease model, particularly for Mendelian diseases. Based on previous research, we set the following filtering criteria: (1) variants confined to the exonic region, (2) elimination of synonymous and non-frameshift variants, (3) filtering out of variants present in the 1000 Genomes Project, ExAC, ESP6500, and gnomAD databases, (4) removal of variants in dbSNP version 130, (5) Combined Annotation-Dependent Depletion phred (CADD-phred) score >10. After identifying the disease variants, we applied the associated genes to the DAVID web tool (http://david.abcc.ncifcrf.gov (accessed on 9 February 2021)) for a functional enrichment analysis of genes with a variety of biological parameters, such as gene ontology (GO), pathways, disease genes, and transcription factor-binding sites. The enrichment test is a statistical analysis that tests the over-representation of genes associated with a functional category in a set of genes. The enrichment test provides information on the functional implications of a set of genes using biological knowledge and statistical testing. We used the enrichment test to determine whether the biological implication of the variants was consistent with the pathophysiology of KTS.

### 2.2. WGS Results

In total, 987,625,204 reads were generated from the processed DNA sample. The proportion of reads with a quality score greater than 20 (>Q20) was 95.64%. The mean depth of the mapped reads was 44.3, and 98.7% of reads had ≥5× coverage.

### 2.3. Results of Annotation Using wANNOVAR and DAVID

Among the 3.9 million single-nucleotide variants (number of single-nucleotide variants = 3,972,786) genotyped by WGS, there were 22,026 variants in exonic regions. We first annotated the exonic variants and applied an enrichment analysis. We excluded synonymous (*n* = 11,004) and non-frameshift substitution (*n* = 142) variants from further analysis. The remaining variants were filtered using our criteria, and there were 34 variants in the results (Supplementary Material Table S1). There were 33 non-synonymous and one stop-gain variants. These variants were mapped to 34 genes and used for an enrichment analysis. An enrichment analysis showed that the ‘cell fate commitment’ biological process GO term was significant, indicating that associated genes were over-represented among the variants (Supplementary Material Table S2). The nominal significance of the enrichment test with this GO term was very strong (*p* = 3.12 × 10^−5^), and the multiple testing correction by the Benjamini-Hochberg method was also significant (*p* = 0.03). The definition of the ‘cell fate commitment’ term is the commitment of cells to specific cell fates and their capacity to differentiate into specific cell types (https://www.ebi.ac.uk/QuickGO/term/GO:0045165 (accessed on 9 February 2021)). Table 1 shows the variants that are members of the ‘cell fate commitment’ GO term.

We also applied the same analysis to non-exonic variants of whole genomes, except for those in intronic and intergenic regions. The contribution of variants in the intronic and intergenic regions has not been widely reported compared with exonic regions in the analysis of genetic diseases, and we excluded variants from these regions. Thereafter, we removed the CADD score criterion because this system has limited clinical validity for variants in the non-coding region. After filtering, 362 variants and 372 genes were mapped and used as input gene lists for enrichment analysis (Supplementary Material Table S3). An enrichment analysis revealed no significant GO terms. Whereas the nominal *p* values showed significance, *p* values based on multiple testing corrections were not significant (Supplementary Material Table S4). However, the nominally significant GO terms were found to be related to development and differentiation terms, such as ‘skeletal system morphogenesis’, ‘embryonic morphogenesis’, and ‘sensory organ development’.

## 3. Discussion

KTS is a rare overgrowth disorder that is genetically caused by a somatic mutation and is typically not inherited. In terms of genes, the most commonly involved mutations are in *PIK3CA* [4]. *PIK3CA* encodes the catalytic subunit of the enzyme phosphatidylinositol 3-kinase, which plays important roles in multiple signaling pathways, such as the vascular endothelial growth factor, fibroblast growth factor, and insulin-like growth factor pathways [5]. These genetic changes are responsible for the development of tissues in the body, resulting in overgrowth and explaining the hyperplasia seen in KTS.

A *PIK3CA* single gene test was reported for KTS [4,6] for determining a targeted molecular diagnosis through a biopsy of affected tissues [3,7]. Luks et al. found that *PIK3CA* mutations were present at low frequencies (<10%) in many affected tissue samples [6]. Due to the inaccessibility or invasiveness of a solid biopsy, next-generation sequencing of cell-free DNA has recently been used as a non-invasive diagnostic tool for KTS [3]. In this study, we performed WGS to explain the difficulty in enriching only *PIK3CA* mutations and affected tissue from biopsies. We detected no mutations in *PIK3CA* by WGS. The use of the GO database maximized the information available for the gene enrichment analysis [8]. The results of the enrichment test with GO terms in exonic regions indicated that the detected variants were causal alleles because the cell fate commitment GO term is related to cellular differentiation, and KTS has features of dysregulated cellular differentiation and development. The significant GO terms were also found to be related to development and differentiation terms in intronic and intergenic regions. These results indicated that variants in non-coding regions were also responsible for genetic diseases, although more evidence that directly links the non-coding variants to phenotypic expression is required to confirm the pathologic non-coding variants.

In terms of clinical manifestations, most reports have focused on bone and soft tissue involvement. Genitourinary involvement was reported in 9–30% of cases, and vulvar hemangioma is the most common gynecologic manifestation [1,9]. A six-year-old female patient with KTS with bloody vaginal discharge and swelling in the left inguinal region was reported [10]. One report showed that five of 218 patients had severe HMB, and all had vascular abnormalities of the uterus and upper vagina on MRI [1]. In this case, the patient also complained of aggravating HMB with the progressive bulging of the clitoris and anterior vaginal wall mimicking pelvic organ prolapse.

A report described that HMB associated with KTS was controlled by luteinizing hormone-releasing hormone analogues and progestogens [11]. However, another patient was unresponsive to a gonadotropin-releasing hormone receptor agonist with add-back therapy and bilateral uterine artery embolization; thus, she underwent a hysterectomy for definitive surgical therapy [12]. Clinicians should also consider the risk of thrombosis, as well as bleeding, in the management of KTS. Jacob et al. reported that nine of 252 patients (4%) with KTS had a pulmonary embolism associated with pelvic surgical procedures, oral contraceptives, pregnancy, and invasive procedures [7]. Therefore, the use of oral contraceptives or antifibrinolytic agents should be avoided in patients with KTS for controlling HMB, and NSAIDs can be the treatment choice, as in the current case. The endometrium with HMB has higher levels of prostaglandins compared with that of normal menstruation [13]. Prostaglandins are vasodilatory agents known to increase cyclooxygenase enzyme expression [14], and thus the administration of cyclooxygenase enzyme inhibitors has been used as therapy for HMB. NSAIDs inhibit cyclooxygenase enzyme systems and reduce menstrual blood loss by 25–35% in around three-quarters of women with HMB [15].

Cutaneous capillary malformation and lymphatic malformation can be also complicated by bleeding and infection [7]. Treatment must be conservative when managing coexisting cellulitis or lymphangitis with antibiotics and compression therapy, as invasive procedures may cause serious bleeding. Husmann et al. reported that patients with cutaneous abnormalities over the trunk or perineum are three-fold more likely to have visceral vascular anomalies [1]. This suggests that port-wine stains of the perineum are predictive of visceral genitourinary involvement. A radiologic evaluation is required to screen patients with KTS for visceral vascular malformations, particularly before major procedures or operations. In our case, sonography and MRI revealed an extensive cavernous hemangioma in the bladder and rectum. Therefore, physicians must be cautious when performing invasive diagnostic exams, such as cystoscopy or recto-sigmoidoscopy, in which malformed vasculature has been noted on imaging to ensure that bleeding from the malformed vasculature is not encountered.

## 4. Conclusions

This is the first report of the involvement of clitoral and vaginal manifestations of KTS mimicking pelvic organ prolapse. The need for a postnatal gynecologic evaluation of the abdominopelvic extension of KTS can be determined, and the risk of bleeding, as well as thrombosis, should be considered in the management of KTS. Further studies are needed to determine the genetic causes of KTS.

## Figures and Tables

**Table 1:** Members of the gene ontology (GO) term “cell fate commitment”.

Chr	Pos	Ref	Alt	RefGene	Amino Acid Change	CADD_Phred
7	39247040	C	T	POU6F2	NM_001166018:exon5:c.C332T:p.P111L,POU6F2:NM_007252:exon5:c.C332T:p.P111L	28.7
11	78498012	C	T	TENM4	NM_001098816:exon16:c.G2296A:p.E766K	24.1
17	7749575	G	C	KDM6B	NM_001080424:exon6:c.G416C:p.S139T,KDM6B:NM_001348716:exon6:c.G416C:p.S139T	22.9
19	1469116	G	C	APC2	NM_001351273:exon14:c.G5813C:p.R1938P,APC2:NM_005883:exon15:c.G5816C:p.R1939P	23.5
21	34400126	C	G	OLIG2	NM_005806:exon2:c.C956G:p.T319S	13.52
22	19754382	C	G	TBX1	NM_080647:exon9:c.C1480G:p.P494A	26.4

GO, Gene Ontology; Chr, Chromosome; Pos, Position; Ref, Reference allele; Alt, Alternative allele; RefGene, Reference gene; CADD_phred, Combined Annotation-Dependent Depletion_phred.

## Data Availability

The data presented in this study are available on request from the corresponding author. The data are not publicly available due to privacy and ethical concerns.

## Supplementary Materials

The following are available online at https://www.mdpi.com/article/10.3390/medicina57040366/s1, Table S1: The results of ANNOVAR annotation in exonic regions, Table S2: Result of DAVID functional annotation, Table S3: Result of ANNOVAR annotation in non-exonic regions, Table S4. Result of Gene Ontology annotation with biological function (BP) terms.
